# Development of an imaging system for visualization of Ebola virus glycoprotein throughout the viral lifecycle

**DOI:** 10.3389/fmicb.2022.1026644

**Published:** 2022-11-03

**Authors:** Wakako Furuyama, Miako Sakaguchi, Kento Yamada, Asuka Nanbo

**Affiliations:** ^1^Department of Virus Infection Dynamics, National Research Center for the Control and Prevention of Infectious Diseases, Nagasaki University, Nagasaki, Japan; ^2^Central Laboratory, Institute of Tropical Medicine (NEKKEN), Nagasaki University, Nagasaki, Japan

**Keywords:** Ebola virus, glycoprotein, transcription- and replication-competent virus-like particle, intracellular traffic, bio-imaging

## Abstract

Ebola virus (EBOV) causes severe EBOV disease (EVD) in humans and non-human primates. Currently, limited countermeasures are available, and the virus must be studied in biosafety level-4 (BSL-4) laboratories. EBOV glycoprotein (GP) is a single transmembrane protein responsible for entry into host cells and is the target of multiple approved drugs. However, the molecular mechanisms underlying the intracellular dynamics of GP during EBOV lifecycle are poorly understood. In this study, we developed a novel GP monitoring system using transcription- and replication-competent virus-like particles (trVLPs) that enables the modeling of the EBOV lifecycle under BSL-2 conditions. We constructed plasmids to generate trVLPs containing the coding sequence of EBOV GP, in which the mucin-like domain (MLD) was replaced with fluorescent proteins. The generated trVLP efficiently replicated over multiple generations was similar to the wild type trVLP. Furthermore, we confirmed that the novel trVLP system enabled real-time visualization of GP throughout the trVLP replication cycle and exhibited intracellular localization similar to that of wild type GP. In summary, this novel monitoring system for GP will enable the characterization of the molecular mechanism of the EBOV lifecycle and can be applied for the development of therapeutics against EVD.

## Introduction

Ebola virus (EBOV) is a highly pathogenic virus that causes Ebola virus disease (EVD) in humans and non-human primates with a fatality rate of up to 90% (Feldmann and Geisbert, 2011). The 2013–2016 EBOV epidemic in West Africa resulted in 11,310 fatalities and 28,616 cases (WHO, 2016; Coltart et al., 2017). The second largest EBOV outbreak, which began in 2018 in the Democratic Republic of the Congo, led to 3,481 cases and 2,299 deaths (WHO, 2021). These outbreaks have accelerated efforts to develop antiviral strategies, and experimental therapeutic and vaccine candidates have been evaluated in clinical trials (Furuyama and Marzi, 2019; Liu et al., 2022; Sivanandy et al., 2022). Moreover, numerous studies have been devoted to therapeutics against EVD, such as monoclonal antibodies and small molecule inhibitors, and considerable progress has been made (Fabozzi et al., 2011; Oestereich et al., 2014; Wolf et al., 2015; Sissoko et al., 2016; Furuyama and Marzi, 2019; Liu et al., 2022; Sivanandy et al., 2022).

EBOV is a prominent member of the *Filoviridae* family of enveloped viruses, with a single-stranded, negative-sense RNA genome of ~19 kb. The EBOV genome encodes seven distinct genes, such as nucleoprotein (NP), polymerase cofactor (VP35), matrix protein (VP40), glycoprotein (GP), transcription activator (VP30), minor matrix protein (VP24), and RNA-dependent RNA polymerase (L), from which at least nine proteins are expressed (Baseler et al., 2017). EBOV expresses three different proteins from its GP gene, GP, soluble GP (sGP), and small soluble GP (ssGP), the expression of which is partially controlled by transcriptional editing at a site of seven uridine residues (7 U; Volchkov et al., 1995; Sanchez et al., 1996; Mehedi et al., 2011). Importantly, a single transmembrane GP is responsible for both receptor binding and membrane fusion; thus, it is the only known target of neutralizing antibodies. EBOV GP undergoes proteolytic cleavage by host proteases such as furin in the trans-Golgi network (TGN), resulting in two subunits, GP1 and GP2 (Volchkov et al., 1998; Jeffers et al., 2002). The GP1 subunit contains a core glycoprotein, receptor-binding domain, glycan cap, and mucin-like domain (MLD). The GP2 subunit contains an internal fusion loop, heptad repeats 1 and 2, a transmembrane region, and a cytoplasmic tail (Sanchez et al., 1996).

EBOV is classified as a biosafety level 4 (BSL-4) agent, which significantly hinders studies on EBOV biology and development of countermeasures. To study the lifecycle of EBOV under BSL-2 conditions, a tetracistronic transcription- and replication-competent virus-like particle (trVLP) system was developed (Hoenen et al., 2014). The tetracistronic minigenome encodes EBOV structural genes such as VP40, VP24, and GP. The expression of the minigenome, along with ribonucleoprotein complex (RNP) proteins, including NP, VP30, VP35, and L in mammalian cells, results in the formation of progeny trVLPs. trVLPs comprise nucleocapsids that contain and release the minigenome into target cells upon infection. Exogenous expression of RNP proteins in target cells leads to another round of minigenome replication and transcription, followed by the formation of infectious trVLPs. Thus, this system allows modeling of the EBOV lifecycle over multiple replication cycles. Originally, the minigenome encoded either luciferase or green fluorescent protein (GFP; Schmidt et al., 2018), serving as a reporter gene. In this study, we improved this system to visualize GP in real time throughout the trVLP lifecycle. This novel trVLP system may provide insights into the molecular mechanisms of intracellular GP trafficking, thus enabling the development of antivirals that target viral entry and assembly processes.

## Materials and methods

### Cells culture

African green monkey kidney epithelial Vero-E6 cells, human embryonic kidney HEK293 cells, HEK293T cells, and human hepatoma Huh7 cells (American Type Culture Collection, VA, United States) were grown in Dulbecco’s modified Eagle’s medium (DMEM; Wako Pure Chemical, Osaka, Japan) containing 10% fetal bovine serum (FBS; Sigma-Aldrich, MO, United States), 100 U/ml penicillin, and 100 μg/ml streptomycin (Wako Pure Chemical). Expi293F cells (kindly gifted by Dr. Kentaro Yoshii, Nagasaki University) were maintained in Expi293 expression medium (Thermo Fisher Scientific, MA, United States), according to the manufacturer’s instructions.

### Plasmids

**Figure 1:**
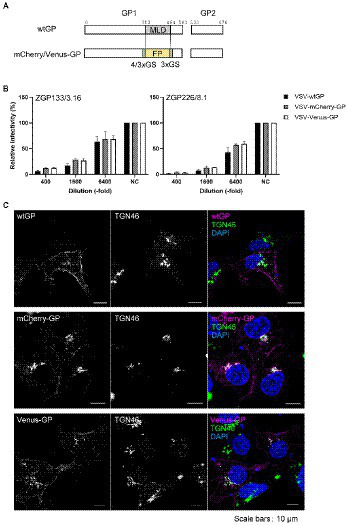
Fluorescent protein-fused GP retained its function and intracellular distribution. **(A)** Schematic representation of fluorescent protein-fused GP. MLD, Mucin-like domain; FP, fluorescent protein; GS; glycine-serine linker. Amino acid positions for the MLD are shown. **(B)** Neutralization assay of the VSV pseudotyped with fluorescent protein-fused GP. VSV pseudotyped with the indicated GPs were incubated with monoclonal antibodies, ZGP133/3.16 or ZGP226/8.1, followed by inoculation into Vero E6 cells. The relative infectivity is shown by setting the IU in the absence of neutralizing antibody. The mean and standard deviation of three independent experiments are shown. **(C)** Intracellular distribution of wild-type, mCherry-, or Venus-fused GP. HEK293 cells were transfected with expression plasmids for wtGP (top), mCherry-GP (middle), or Venus-GP (bottom). The cells were harvested at 24 hpt and distribution of individual GP derivatives (left, magenta in the merged images) and TGN46 (middle, green in the merged images) were analyzed by immunofluorescence staining. The nuclei were counterstained with DAPI (blue in the merged images). Scale bars: 10 μm.

The mCherry or Venus-fused EBOV GP gene (mCherry-GP or Venus-GP) was generated by replacing the MLD with mCherry (Nanbo et al., 2018) or Venus (kindly provided by Dr. Takeharu Nagai, Osaka University) cDNAs separated with a 3 or 4× glycine-serine (GS) linker at both ends (Figure 1A) using an In-Fusion Cloning kit (TaKaRa Bio, Kusatsu, Japan). Each gene was inserted into an expression plasmid (pCAGGS) or tetracistronic minigenome plasmid with a luciferase gene (p4cis-vRNA-Luc). mCherry-GP and Venus-GP inserted into p4cis-vRNA-Luc were mutated from 8A to 7A at the editing site using a PrimeSTAR Mutagenesis Basal Kit (TaKaRa Bio). The detailed cloning strategies are available upon request. The expression plasmids for the RNP proteins T7 and p4cis-vRNA-Luc were kindly provided by Dr. Andrea Marzi (NIH/NIAID, Rocky Mountain Laboratories, United States). An expression plasmid for a GFP-fused Golgi marker (pAc-GFP-Golgi) was obtained from TaKaRa Bio.

### Generation of vesicular stomatitis virus pseudotyped with fluorescent proteins-fused GP

The replication-incompetent vesicular stomatitis virus (VSV) pseudotyped with wild-type EBOV GP (wtGP), mCherry-GP, or Venus-GP containing GFP instead of the VSV glycoprotein (G) gene (VSV-wtGP, VSV-mCherry-GP, or VSV-Venus-GP) was generated as described previously (Takada et al., 1997, 2003). Briefly, HEK293T cells were transfected with mCherry-GP or Venus-GP expression plasmids. At 24 h post-transfection (hpt), the cells were infected with pseudotyped VSV possessing G (VSV-G) at a multiplicity of infection of more than 1. At 18 h post-infection (hpi), supernatants were harvested and centrifuged at 3,500 rpm for 15 min at room temperature to remove cell debris. Virus titers in the supernatants were determined using Vero-E6 cells as described previously (Takada et al., 1997).

### Neutralizing assay

VSV-wtGP, VSV-mCherry-GP, and VSV-Venus-GP were diluted to yield 2,000–3,500 infectious units (IUs). They were preincubated with diluted neutralizing antibodies against EBOV GP (ZGP133/3.16 or ZGP226/8.1; kindly provided by Dr. Ayato Takada, Hokkaido University, Japan) and VSV-G (VSV-G[N]1–9; kindly provided by Dr. Takada) to reduce the background derived from infection with VSV-G for 1 h at room temperature. The mixture was then inoculated into 2.5 × 10^4^ cells/well Vero E6 cells grown in 96-well plates with DMEM containing 5% FBS. At 24 hpi, the number of GFP-positive cells was counted by fluorescence microscopy, and the relative percentage of infectivity was measured by setting the number of infected cells in the absence of the neutralizing antibody to 100%.

### Immunofluorescence staining

HEK293 cells grown on 35 mm glass-bottom dishes (MatTek Corporation, OR, United States) were transfected with the expression plasmids for wtGP, mCherry-GP, or Venus-GP. At 24 hpt, the cells were fixed with 4% paraformaldehyde (PFA; Electron Microscopy Sciences, PA, United States) in PBS for 15 min at room temperature, permeabilized with PBS containing 0.05% Triton X-100 (Nacalai Tesque Inc., Kyoto, Japan) for 10 min at room temperature, and blocked with 4% bovine serum albumin (BSA; Merck, Frankfurt, Germany) in PBS for 30 min at room temperature. The cells were incubated with a mouse anti-EBOV GP monoclonal antibody (ZGP12/1.1, 5 μg/ml; kindly provided by Dr. Takada), and a rabbit anti-TGN46 polyclonal antibody (Novus Biologicals, CO, USA, 1:400 dilution) for 1 h at room temperature. The cells were washed with PBS and incubated in the presence of Alexa Fluor 488- (Abcam, Cambridge, United Kingdom), 594- (Thermo Fisher Scientific), or 647- (Thermo Fisher Scientific) labeled secondary antibodies (1,400 dilution) for 1 h at room temperature. After washing, the nuclei were counterstained with 4′,6-diamidino-2-phenylindole dihydrochloride (DAPI, 1 μg/ml; Thermo Fisher Scientific). Images were collected with a 60 × oil-immersion objective lens of a confocal laser scanning microscope (FV3000, Olympus, Tokyo, Japan) and acquired using the FV31S-SW software (Olympus).

### Purification of trVLPs and EBOV-like particles (Ebola VLPs)

To produce trVLPs, HEK293 cells were transfected with plasmids encoding T7-polymerase, a tetracistronic minigenome, and RNP expression plasmids, as described previously (Biedenkopf and Hoenen, 2017). To produce Ebola VLPs, Expi293F cells were transfected with equal amounts of expression plasmids encoding EBOV GP, VP40, and NP. At 72 hpt, the culture supernatants were harvested and centrifuged at 3,500 rpm for 15 min at room temperature. trVLPs and VLPs were precipitated through a 25% sucrose cushion by centrifugation at 11,000 rpm for 1 h at 4°C using a JS-24 38 rotor (Beckman Coulter, CA, United States). Precipitated trVLPs and VLPs were suspended in TNE buffer [10 mM Tris–HCl (pH 8.0), 100 mM NaCl, and 1 mM EDTA] overnight at 4°C and fractionated through a 20%–50% sucrose gradient in PBS at 24,000 rpm with a JS-24 15 rotor (Beckman Coulter) for 2 h at 4°C. Subsequently, the trVLP and VLPs fractions were sedimented by ultracentrifugation at 11,000 rpm for 1 h at 4°C with a JS-24 38 rotor. Finally, the trVLPs and VLP pellets were resuspended in TNE buffer and incubated overnight at 4°C. The protein content in each fraction was measured using the Bradford protein assay kit (Bio-Rad, Hercules, CA, United States).

### EBOV trVLP assay

trVLPs were produced in HEK293 cells, and a trVLP assay was performed using Huh7 cells, as described previously (Biedenkopf and Hoenen, 2017), with the indicated modifications. HEK293 cells grown in 6-well plates were transfected with plasmids encoding T7-polymerase, a tetracistronic minigenome, and expression plasmids for NP, VP35, VP30, and L. Seventy-two hours later, trVLPs in the supernatant were harvested and centrifuged at 3,500 rpm for 10 min at 4°C to remove cell debris. trVLPs were infected into HEK293 cells 4 h after pre-transfection with RNP protein expression plasmids. Since the target cells (P1–P5) of the trVLP assay were sensitive to being left without a medium, the trVLP infection was performed one well at a time. Seventy-two hours after treatment, cell lysates were prepared using the Dual-Luciferase Reporter Assay System (Promega, Walldorf, Germany), according to the manufacturer’s protocol, and luminescence was measured using a SpectraMax iD5 microplate reader (Molecular Devices, CA, United States).

### Western blot analysis

trVLPs and Ebola VLPs were incubated in sodium dodecyl sulfate-polyacrylamide gel electrophoresis (SDS-PAGE) sample buffer at 95°C for 5 min, followed by western blotting using mouse monoclonal antibodies against EBOV GP (ZGP 42/3.7, 1 μg/ml; kindly provided by Dr. Takada), VP40 (cl. 6, kindly provided by Dr. Yoshihiro Kawaoka, University of Wisconsin-Madison, 1:1,000 dilution), or NP (cl. 7–71.5, kindly provided by Dr. Kawaoka, 1:1,000 dilution).

### Negative stain electron microscopy

The trVLPs and Ebola VLPs were fixed with 4% PFA in PBS at 4°C overnight. Each sample was loaded onto a 200-mesh copper grid with a carbon-coated plastic film (Nisshin EM, Tokyo, Japan) immediately following glow discharge and negatively-stained with uranyl acetate solution (1%, w/v) for 15 s. The morphology of each sample was observed using JEM-1400Flash (JEOL, Tokyo, Japan) with an acceleration voltage of 80 kV.

### Visualization of intracellular distribution of GP in trVLP-infected cells

HEK293, Vero-E6, or Vero-E6 cells stably expressing enhanced GFP-fused to Rab7 (eGFP-Rab7; Nanbo et al., 2010) were cultured in 35 mm glass-bottom dishes. HEK293 cells were co-transfected with tetracistronic minigenome plasmids encoding wtGP, mCherry-GP, or RNP. At 48 hpt, the cells were fixed with 4% PFA, and the distribution of GP and TGN was analyzed by immunofluorescence staining as described previously. Vero-E6 or Vero-E6 eGFP-Rab7 cells pre-transfected with expression plasmids for RNP proteins were inoculated with purified trVLPs (2 μg/ml) containing wtGP- or mCherry-GP-encoded minigenomes by incubation for 30 min on ice. The cells were incubated in DMEM containing 2% FBS and 4% BSA for 0, 2, or 48 h at 37°C. At the indicated time points, the cells were fixed with 4% PFA and the distribution of GP, Rab7, or TGN (48 h) was analyzed by immunofluorescence staining. To analyze the colocalization of GP and TGN in live cells, HEK293 cells were cotransfected with pAc-GFP-Golgi, tetracistronic minigenome, and RNP plasmids. Vero-E6 cells pre-transfected with the pAc-GFP-Golgi and RNP plasmids were inoculated with 2 μg/ml trVLPs. At 48 hpt, the distribution of GP and TGN in these cells was analyzed as described previously (Bhattacharyya and Hope, 2011). Nuclei were counterstained with 1 μg/ml Hoechst 33342 (Cell Signaling Technology, MA, United States) or DAPI. Images were collected with the 60 × oil-immersion objective lens of a confocal laser scanning microscope and acquired using the FV31S-SW software.

## Results

### Generation of fluorescent protein-fused EBOV GPs

Previous studies have demonstrated that replication-competent VSV pseudotyped with EBOV GP lacking the MLD could infect and propagate in cells in a manner similar to that of VSV possessing wtGP (Zhang et al., 2020; Bhatia et al., 2021). Furthermore, a previous study established a visualization system for GP of recombinant Marburg virus, a member of the *Filoviridae* family, by replacing MLD with a fluorescent protein (Mittler et al., 2018). Thus, in this study, we constructed two expression plasmids containing the coding sequence of EBOV GP, in which MLD was replaced with fluorescent proteins, such as mCherry (Shaner et al., 2004) or Venus (Nagai et al., 2002; Figure 1A). MLD constitutes part of GP and contains dense clusters of sites predicted to be modified by mucin-type O-glycosylation (Lee et al., 2008). The VSV-based pseudotyped virus system has been well established and used in previous studies to investigate GP-mediated EBOV entry into cells (Takada et al., 1997). We also examined the effect of replacing the MLD with fluorescent proteins on the viral entry process of replication-incompetent VSV pseudotyped with mCherry- or Venus-fused GP (VSV-mCherry-GP or VSV-Venus-GP). We found that VSV containing both GP derivatives could infect Vero-E6 cells in a manner similar to that of VSV possessing wild-type GP (VSV-wtGP; Figure 1B). In addition, two independent neutralizing antibodies, ZGP133/3.16 and ZGP 226/8.1 (Marzi et al., 2012), inhibited the infection of VSV pseudotyped wtGP and two GP derivatives in a dose-dependent manner (Figure 1B).

We further characterized the intracellular distribution of the GP derivatives. It is known that the O- and N-glycosylations occur in the TGN (Volchkov et al., 1998). Consistent with previous reports (Bhattacharyya and Hope, 2011; Nanbo et al., 2013), immunofluorescence staining revealed that wtGP (Figure 1C, top) and the two fluorescent protein-fused GP derivatives (Figure 1C, middle and bottom) were predominantly distributed in the perinuclear region and partially colocalized with TGN46, a TGN marker (Figure 1C, top). Some fractions of wt and fluorescent protein-fused GPs were localized in the plasma membrane, indicating that GP traffics to the cell surface where EBOV particles bud. These results suggest that replacement of MLD with fluorescent proteins does not affect the intracellular distribution of EBOV.

### Effect of replacing fluorescent protein-fused GPs with MLD on EBOV particle formation

**Figure 2:**
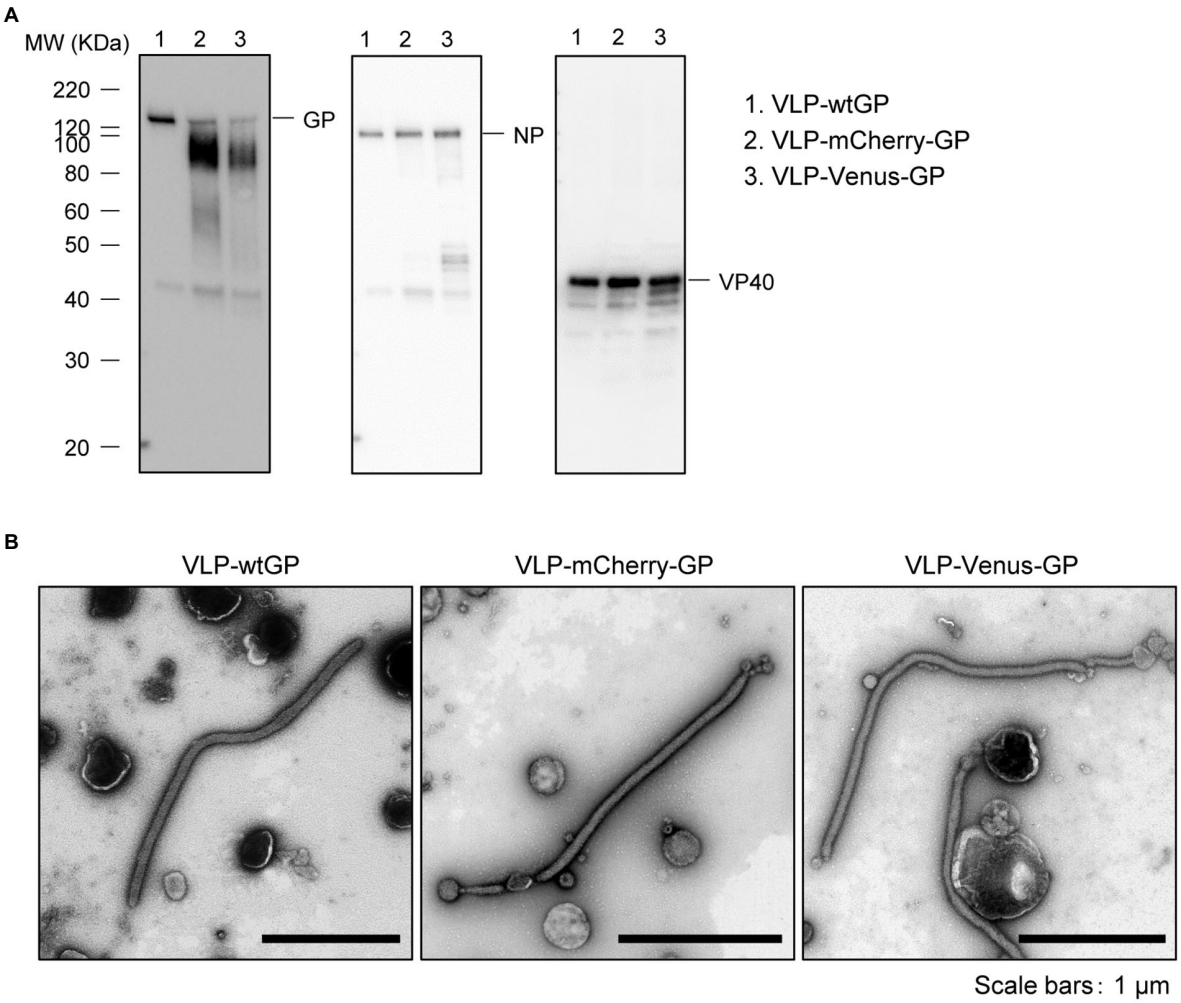
Morphological properties of Ebola VLPs containing fluorescent protein-fused GPs. **(A)** Validation of incorporation of fluorescent protein-fused GPs into viral particles. Expi293F cells were transfected with the expression plasmids for wt and two GP derivatives, NP and VP40. Ebola VLPs released into culture supernatants were purified by ultracentrifugation and expression of GP (left), NP (middle), and VP40 (right) in purified VLPs was analyzed by western blot analysis. **(B)** Analysis of morphological features of VLPs containing fluorescent protein-fused GPs. Purified VLPs spiked with wtGP (left), mCherry-GP (middle) and Venus-GP (right) were analyzed by negative staining with electron microscopy. Scale bars: 1 μm.

To confirm that fluorescent protein-fused GP does not affect EBOV particle formation and its incorporation into EBOV particles, we produced Ebola VLPs by expressing the major EBOV structural proteins GP, VP40, and NP in HEK293T cells. The incorporation of mCherry- or Venus-GP, along with NP and VP40, into the VLPs released in the culture medium was confirmed by western blot analysis (Figure 2A). We also analyzed the morphological properties of individual VLPs by transmission electron microscopy (TEM). Ebola VLPs containing both fluorescent protein-fused GPs (Figure 2B, middle and right) presented filamentous morphologies with surface spikes, similar to those of VLPs containing wtGP (Figure 2B, left). These data indicated that the replacement of these fluorescent proteins with MLD did not negatively affect the incorporation of GP into the viral particles or viral particle formation and morphology.

### Establishment of EBOV trVLPs encoding a fluorescent protein-fused GP gene

**Figure 3:**
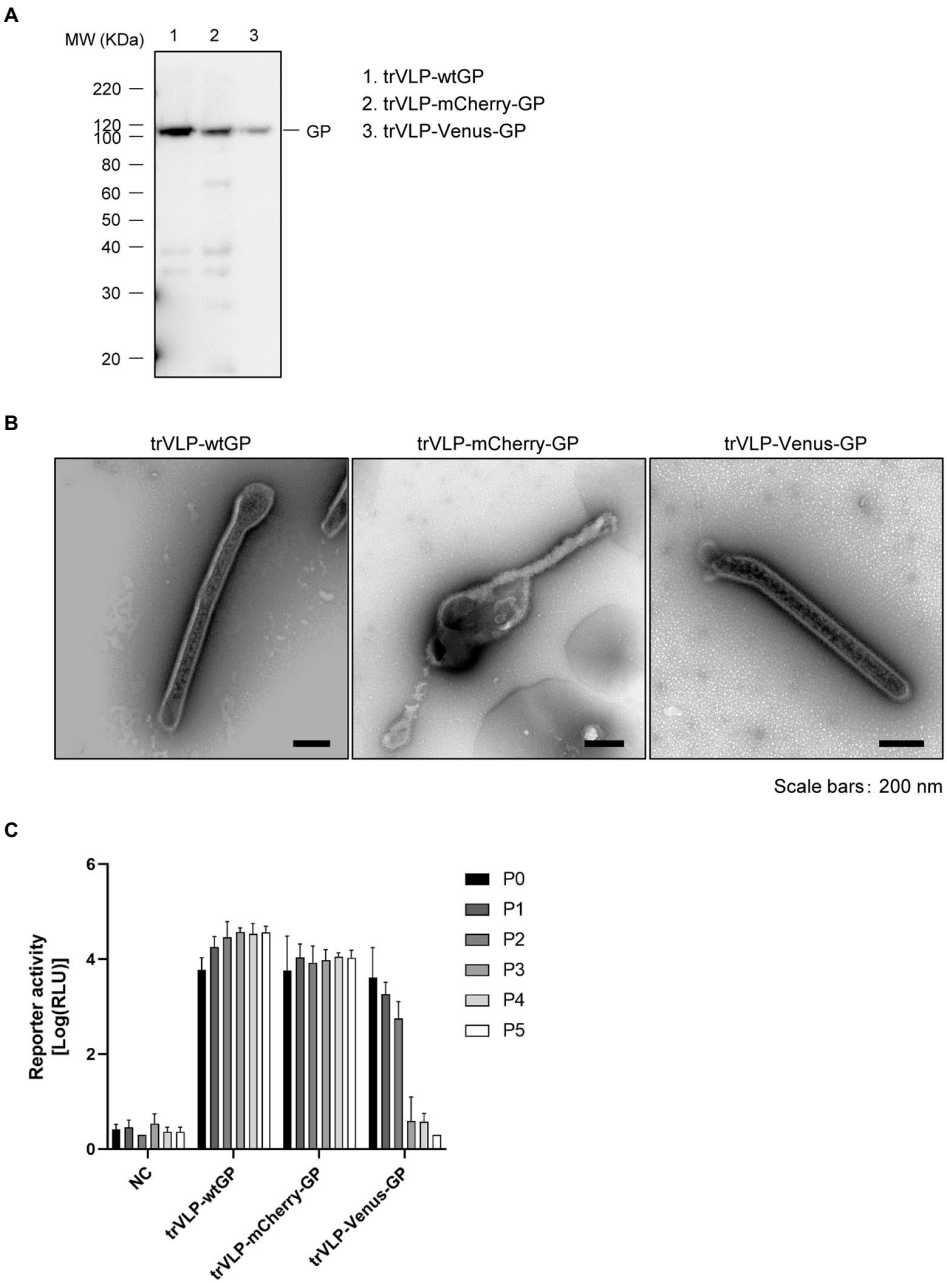
Establishment of EBOV trVLPs encoding the fluorescent protein-fused GP **(A)** Incorporation of the fluorescent protein-fused GP into the tetracistronic trVLPs. trVLP-wtGP, -mCherry-GP, or -Venus-GP released into culture supernatant of transfected-HEK293 cells (P0) were purified by ultracentrifugation. Incorporation of GP derivatives into purified trVLP was determined by western blot analysis. **(B)** Confirmation of morphology of tetracistronic trVLPs encoding the fluorescent protein-fused GP. Purified trVLP-wtGP (left), trVLP-mCherry-GP (middle), or trVLP-Venus-GP (right) were visualized by negative staining. Scale bars: 200 nm. **(C)** Reporter activities of trVLPs possessing a tetracistronic minigenome that encodes fluorescent protein-fused GP throughout multiple generations. Huh7 cells (P1) pre-transfected with expression plasmids encoding the RNP proteins were inoculated with culture supernatant of transfected HEK293 cells (P0). Seventy-two hpi, reporter activities were measured using the Dual-Luciferase Reporter Assay System. This infection was repeated every 72 h for 5 passages (P5). As a negative control, naïve HEK293 cells are shown. The means and standard deviations from 3 independent experiments are shown.

We further characterized fluorescent protein-fused GPs in the context of the trVLP system. We replaced the GP gene in the minigenome with mCherry- or Venus-GP to produce trVLPs in HEK293 cells. The incorporation of these GP derivatives into purified trVLPs and the morphological features of the progeny virions were confirmed by western blotting (Figure 3A) and TEM analyses (Figure 3B), respectively. A previous study demonstrated that tetracistronic trVLPs could pass and produce trVLPs into target cells that had been pre-transfected with expression plasmids encoding viral RNP proteins (Biedenkopf and Hoenen, 2017).

As our next step in characterizing the fluorescent protein-fused GP, including the minigenome system, we assessed whether the trVLPs possessing a minigenome that encodes the fluorescent protein-fused GP undergo multiple replication cycles. Consistent with previous studies (Hoenen et al., 2014), the reporter activity of trVLP-mCherry-GP was readily detectable and remained stable over five passages (P5; Figure 3C). Furthermore, no mutations were observed in the genome until P5 (data not shown), indicating that mCherry-GP-encoded trVLPs are stable for multiple infectious cycles. In contrast, trVLP-Venus-GP showed reporter activity over three passages (Figure 3C) without any mutations in the genome (data not shown). Therefore, trVLP-mCherry-GP was used to develop the GP visualization system.

### Visualization of entry process of trVLPs bearing mCherry-GP

**Figure 4:**
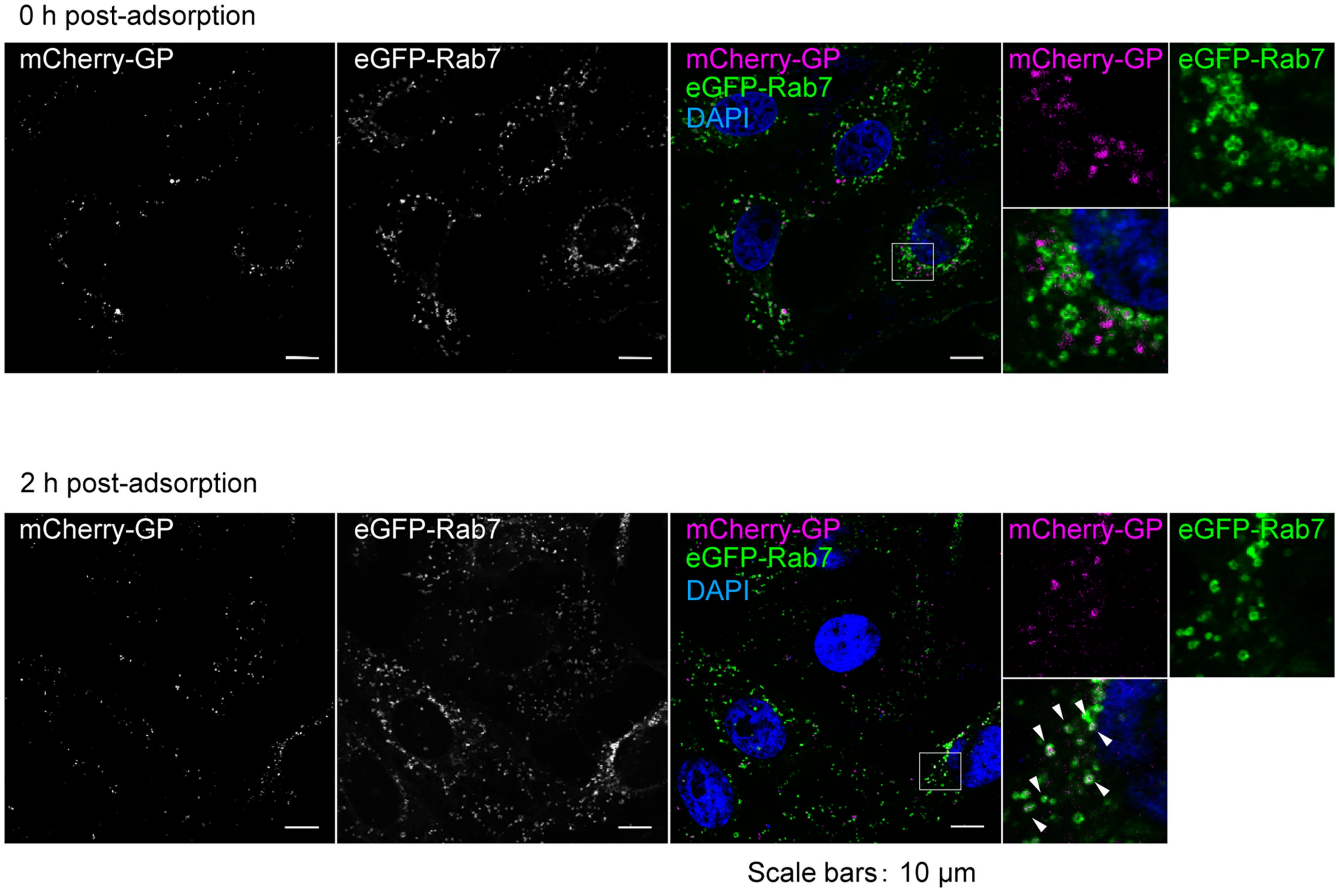
Visualization of entry process of trVLP-mCherry-GP into recipient cells. trVLP-mCherry-GP was inoculated into Vero E6 cells expressing eGFP-Rab7 and incubated for 30 min on ice. After adsorption, cells were incubated for 0 h (top) or 2 h (bottom) at 37°C. The distribution of mCherry signals (left) on the cell surface (0 h, top), in the cytoplasm (2 h, bottom), and in eGFP-Rab7 (middle) was analyzed by confocal laser scanning microscopy. mCherry-GP and eGFP-Rab7 are shown in magenta and green, respectively, in the merged image. The insets show boxed areas. The white arrows represent colocalized signals. The nuclei were counterstained with DAPI (blue in the merged images). Scale bars: 10 μm.

To validate the capacity of trVLP-mCherry-GP to enable the visualization of intracellular GP dynamics, we first examined the entry process. In our previous studies, we demonstrated that fluorescently labeled Ebola VLPs were attached to the cell surface and internalized into the cells *via* macropinocytosis in a GP-dependent manner. Ebola VLPs were then trafficked to Rab7-positive late endosomes 2 h post-inoculation, and the internalized viral envelope was fused to the endosomal membrane 6 h after inoculation (Nanbo et al., 2010; Furuyama et al., 2016a,b). Using trVLP-mCherry-GP, we successfully observed mCherry signals in recipient cells under a laser-scanning confocal microscope (Figure 4). mCherry signals were first detected on the surface of the cells in which trVLPs were adsorbed (Figure 4, top). Moreover, 2 h after adsorption, mCherry signals were visualized as a speckle pattern that predominantly colocalized with eGFP-Rab7 (Figure 4, bottom, white arrows), indicating that internalized trVLPs were trafficked into the late endosomes.

### Visualization of intracellular traffic of GP in trVLPs-infected cells

**Figure 5:**
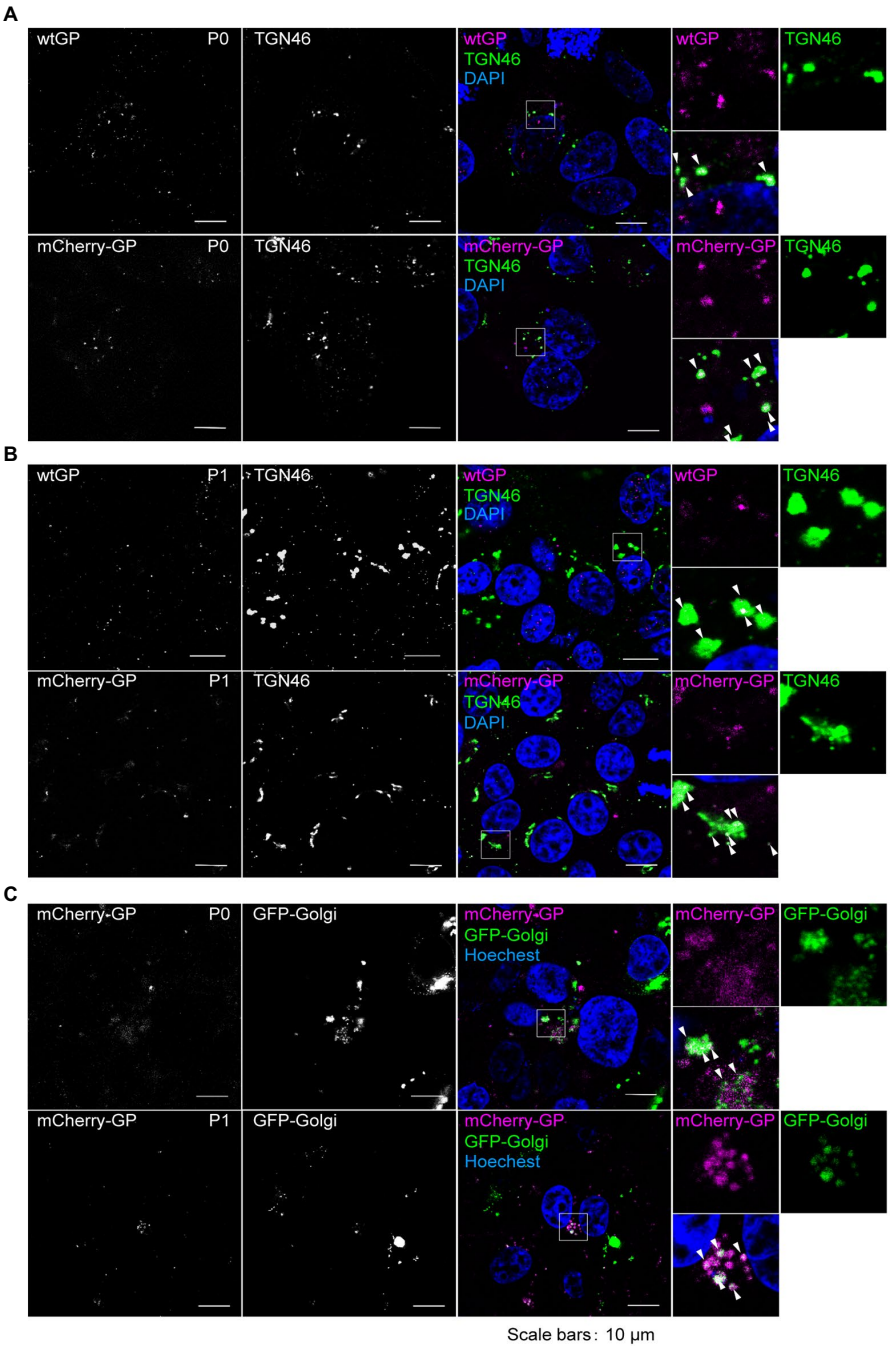
Visualization of intracellular traffic of mCherry-GP in the trVLP-infected cells. **(A)** Distribution of mCherry-GP in HEK293 (P0 cells). HEK293 cells were co-transfected with the plasmid for minigenome and the expression plasmids for T7 and RNP proteins. At 48 hpt, the cells were harvested and intracellular distributions of wtGP (top, magenta in the merged images) or mCherry-GP (bottom, magenta in the merged images) and TGN46 (middle, green in the merged images) were analyzed by immunofluorescence staining with antibodies against GP (for wtGP) or TGN46, respectively. **(B)** Distribution of mCherry-GP in Vero-E6 (P1 cells). Vero-E6 cells pre-transfected with the expression plasmids for the RNP proteins were incubated with trVLP-wtGP (top) or trVLP-mCherry-GP (bottom). Forty-eight hours after inoculation, the cells were harvested and intracellular distributions of wtGP (top) or mCherry-GP (bottom) and TGN46 (middle) were analyzed as described above. **(C)** Live-cell imaging of mCherry-GP in P0 and P1 cells. HEK293 (P0 cells, top) or Vero-E6 cells (P1 cells, bottom) were pre-transfected with an expression plasmid for GFP-Golgi. At 48 hpt, distribution of mCherry-GP was analyzed by confocal laser scanning microscopy. Nuclei (blue) were counterstained with DAPI **(A,B)** or Hoechst 33342 **(C)**. Insets show the enlarged images of boxed areas. Arrows represent co-localized signals. Scale bars: 10 μm.

Next, we characterized the intracellular trafficking of GP during the late stages of the EBOV lifecycle. It was reported that the EBOV GP is transported to the assembly sites through the TGN (Volchkov et al., 1998; Bhattacharyya and Hope, 2011; Nanbo et al., 2013). To test whether replicated mCherry-GP was trafficked through an identical pathway, we transfected HEK293 cells with a series of expression plasmids to produce progeny trVLPs-mCherry-GP (P0). At 48 hpi, the distribution of mCherry-GP in the TGN was analyzed by immunofluorescence staining using an antibody against TGN46, a TGN marker. At 48 hpi, both wtGP (Figure 5A, top) and mCherry-GP (Figure 5A, bottom) were distributed in perinuclear regions and colocalized with TGN46 (Figure 5A, white arrows). We further examined the stability of mCherry-GP distribution in a single replication cycle. In P1 trVLP-producing cells, obtained by inoculation of trVLP mCherry-GP into Vero-E6 cells pre-transfected with expression plasmids for the RNP proteins, mCherry-GP signals exhibited a similar tendency to those observed in P0 cells (Figure 5B). Finally, we applied this system to live cell imaging using Vero-E6 cells pre-transfected with an expression plasmid for GFP-fused Golgi markers. mCherry-GP signals were detected in both P0 and P1 cells (Figure 5C) and colocalized with GFP-Golgi (Figure 5C). These results indicate that we have developed a novel trVLP-based system to visualize GP dynamics throughout the EBOV lifecycle.

## Discussion

In this study, we developed a novel monitoring system for EBOV GP by applying a tetracistronic EBOV trVLP system. We constructed two GP derivatives fused to fluorescent proteins mCherry and Venus by replacing the MLD with individual fluorescent proteins. Both constructs exhibited similar antibody neutralization profiles (Figure 1B) and morphological properties in Ebola VLPs and trVLPs (Figures 2B, 3B) compared with those of wtGP. These derivatives were also capable of mediating the internalization of trVLPs (Figure 4) and their appropriate subcellular distribution (Figures 1C, 5). These results indicate that replacing MLD with fluorescent protein does not affect GP function. Previous studies demonstrated that GP lacking the MLD exhibited smear band, while full-length GP gives a sharp single band (Martinez et al., 2011; Wang et al., 2017). Smear bands were observed by western blotting for mCherry-GP and -Venus-GP derived from Ebola VLPs obtained from Expi293F cells, whereas wtGP exhibited a single band. However, both wt and fluorescent protein-fused GP expressed in the trVLPs generated from HEK293 cells were of the same size (Figure 3A). These results indicate that the glycosylation status of fluorescent protein-fused GP might be different between Expi293F and HEK293 cells. Furthermore, trVLP-Venus-GP exhibited notably reduced reporter activity during the first two replication cycles compared with trVLPs possessing wtGP and mCherry-GP (Figure 3C). In addition, the reporter activity of trVLP-Venus-GP in the third-round passage decreased to background levels, whereas trVLP-mCherry-GP did not exhibit any reduction in activity (Figure 3C). As no mutation in the minigenome of trVLP-Venus-GP was observed until the second-round passage (data not shown), the reduction in luciferase activity might be caused by differences between Venus and mCherry in terms of stability in the cellular environment or efficacy of trVLP propagation. Furthermore, Figure 3A demonstrates that the amount of released trVLPs possessing Venus-GP was lower than that of wtGP and mCherry-GP, suggesting that Venus-GP likely suppresses the budding process of trVLPs. Additional characterization is required to elucidate the mechanisms underlying this phenomenon.

Previous studies have established a monitoring system for the individual processes of EBOV entry, including virus attachment, internalization, and membrane fusion, using lipophilic tracer (DiI)-labeled Ebola VLPs (Nanbo et al., 2010). This system was further applied to analyze the inhibitory mechanisms of monoclonal antibodies and compounds that target EBOV entry (Furuyama et al., 2016b; Isono et al., 2020). The system can even analyze the membrane fusion efficiency by detecting dequenched DiI fluorescence (Nanbo et al., 2010). Although our trVLP systems were unable to analyze the membrane fusion process because the fluorescent protein-fused GP could not be dequenched, they were able to monitor the attachment and internalization of trVLPs (Figure 4).

Mittler et al. established a monitoring system for intracellular traffic of Marburg virus-encoded GP in the context of the recombinant virus and the infectious VLPs which could not replicate multiple cycles. Nevertheless, our system is capable of visualizing the translated GP in the later stages of the EBOV lifecycle under BSL-2 conditions. We demonstrated that mCherry-GP was predominantly distributed in the perinuclear TGN for assembly, which is consistent with previous observations for wild-type GP (Figure 5; Volchkov et al., 1998). Further investigation of GP dynamics in the context of trVLPs will clarify the molecular mechanism of the viral assembly and budding processes in more detail.

Real-time monitoring systems for the intracellular dynamics of VP40 (Adu-Gyamfi et al., 2012; Frick et al., 2015) and nucleocapsid-like structural proteins, including NP, VP35, and VP24 (Takamatsu et al., 2018), in live cells have been developed. Integration with our system enables a more detailed characterization of molecular dynamics in terms of host–virus interactions during the EBOV replication cycle.

Currently, limited pharmacological therapies are available for the treatment and prevention of EVD. GP is the only surface protein of EBOV particles and is responsible for the EBOV entry process. Several pharmacological drugs that inhibit GP-mediated entry processes, including monoclonal antibodies and compounds, have been approved (Liu et al., 2022). However, only a few targeting the GP trafficking pathway have been investigated (Shrivastava-Ranjan et al., 2018). By applying for GP derived from other filovirus family members, our monitoring system will help further understand the molecular mechanisms underlying the intracellular dynamics of GP throughout the viral lifecycle, which may lead to the discovery and development of novel pan-therapeutics against filovirus family.

In conclusion, we established a novel system to monitor the intracellular trafficking of GP using tetracistronic trVLPs. This technique will serve as a useful tool in investigating the molecular mechanism underlying the EBOV lifecycle and support the development of new therapeutics against EVD.

## Data availability statement

The original contributions presented in the study are included in the article/supplementary material, further inquiries can be directed to the corresponding author.

## Author contributions

WF and AN conceived of the study, designed the experiments, secured the funding, analyzed the data, and prepared the manuscript. WF, KY, and MS performed experiments. All authors contributed to the article and approved the submitted version.

## Funding

This research was supported by grants from the Japan Society for the Promotion of Science (21K20762), Japan Agency for Medical Research and Development (JP22fm0208101 and 21461223), Joint Usage/Research Center programs from Hokkaido University Research Center for Zoonosis Control, Institute of Medical Science, University of Tokyo, Institute for Frontier Life and Medical Sciences, Kyoto University, Tokyo Biochemical Research Foundation, Takeda Science Foundation and Medical Research Grants, and Astellas Foundation for Research on Metabolic Disorders. The funders had no role in the study design, data collection and analysis, decision to publish, or manuscript preparation.

## Conflict of interest

The authors declare that the research was conducted in the absence of any commercial or financial relationships that could be construed as a potential conflict of interest.

## Publisher’s note

All claims expressed in this article are solely those of the authors and do not necessarily represent those of their affiliated organizations, or those of the publisher, the editors and the reviewers. Any product that may be evaluated in this article, or claim that may be made by its manufacturer, is not guaranteed or endorsed by the publisher.
